# Fistule vesico-sigmoïdienne compliquant une hydatidose intestinale: à propos d'un cas rare

**DOI:** 10.11604/pamj.2014.19.117.5220

**Published:** 2014-10-01

**Authors:** Mounir Lahyani, Younes Jabbour, Tarik Karmouni, Khalid Elkhader, Abdellatif Koutani, Ahmed Ibn attya Andaloussi

**Affiliations:** 1 Service d'Urologie B, CHU Ibn Sina Rabat, Rabat, Maroc

**Keywords:** Fistule vesico-sigmoidienne, hydatidose intestinale, bénigne, Vesico-sigmoid fistula, intestinal hydatidosis, benign

## Abstract

La fistule colo-vésicale sur une hydatidose sigmoidienne est une entité pathologique exceptionnelle. Nous rapportions une nouvelle observation, ou seront rappelées les principales données diagnostique et thérapeutique de cette affection.

## Introduction

Bien qu'il s'agisse d'une lésion habituellement bénigne, le diagnostic des fistules vésico-sigmoïdienne (FVS) n'est pas toujours facile, notamment le diagnostic étiologique pouvant amener à indiquer une laparotomie exploratrice et thérapeutique. A travers notre observation, nous allons mettre en relief les particularités cliniques, paracliniques et thérapeutiques d'un cas exceptionnel, voire même jamais décrit dans la littérature de FVS secondaire à une hydatidose sigmoïdienne.

## Patient et observation

**Figure 1:**
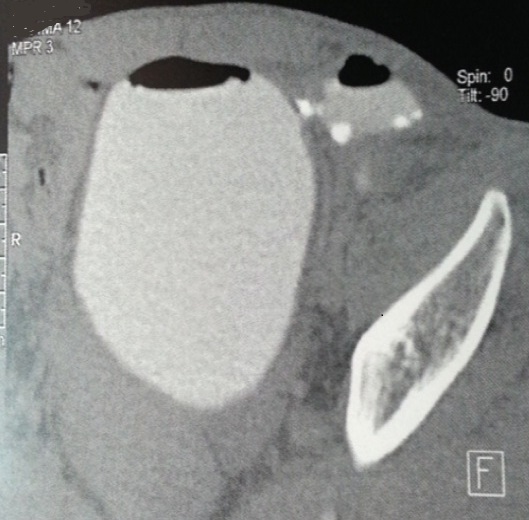
Coupe uro-TDM montrant le trajet fistuleux vésico-sigmoidien et la présence d'air en intra-vésical

**Figure 2:**
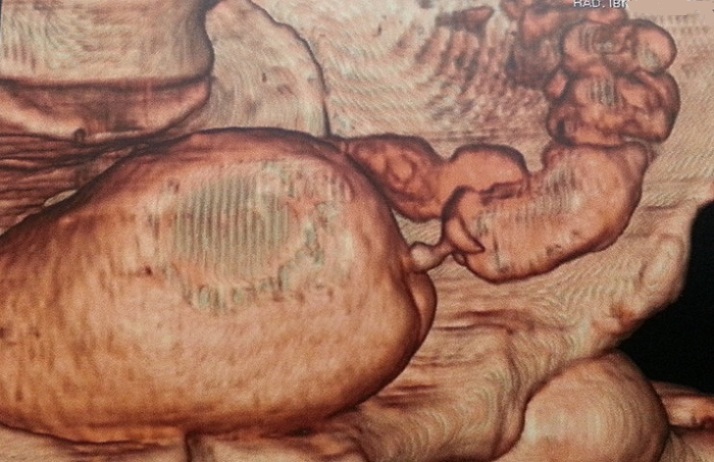
Image scannographique 3D d'une FVS

**Figure 3:**
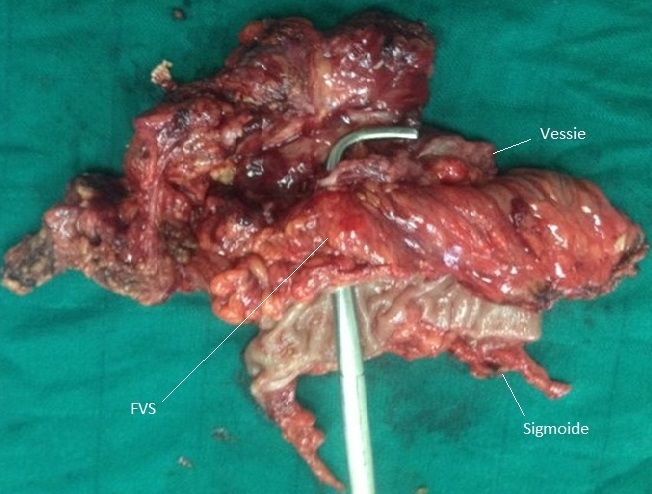
Pièce opératoire après sigmoidectomie et résection de la FVS avec la colerette vésicale

**Figure 4:**
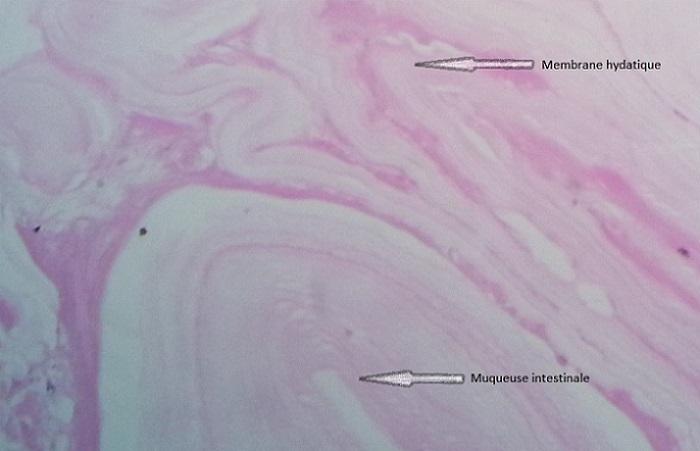
Mise en évidence des membranes hydatiques à l'examen histologique

Mr A.A, âgé de 48 ans, antécédent de kyste hydatique hépatique opéré, est suivi depuis un an pour des diarrhées intermittentes et des signes urinaires irritatifs faites de pollakiurie diurne et nocturne et brûlures mictionnelles sans hématurie pour lesquelles l'enquête étiologique clinique ainsi que paraclinique (cytologie urinaire, examen cytobactériologique des urines, échographie et cystoscopie) était négative. Le patient reconsulte six mois plus tard pour fécaliurie associée à une pneumaturie avec un examen somatique normal, en particulier le toucher rectal qui montre une prostate plate non suspecte. Dans le cadre du bilan étiologique, l'uroscanner a mis en évidence la présence d′air dans la vessie avec un épaississement irrégulier de sa corne gauche ainsi qu′une une fistule entérovésicale opacifiant le sigmoïde sans spécificité étiologique (Figure 1, Figure 2). Ce bilan a été complété par une cystoscopie qui a montré une zone très inflammatoire localisée et située au niveau du dôme vésical; pouvant correspondre à l'orifice fistuleux. Malheureusement, une biopsie à ce niveau est non faite. Une recto-colonoscopie a été réalisée et a objectivé un aspect pseudo-tumoral 5 cm au dessus de la jonction recto-sigmoïdienne, l′examen histologique de la biopsie est revenu en faveur d′un remaniement inflammatoire non spécifique et sans signes de malignité. On décide de pratiquer une laparotomie par une médiane à cheval sur l'ombilic, la libération du sigmoïde ont mis en évidence une gaine fibreuse sur le versant anti-mésocolique de la charnière recto-sigmoidienne fistulisée au niveau du dôme vésical, ce qui a nécessité la résection d'un patch vésical péri fistulaire de 4 cm de diamètre associée à résection segmentaire du sigmoïde et rétablissement immédiat de la continuité digestive (Figure 3). Les suites opératoires étaient simples avec reprise du transit au quatrième jour postopératoire et la sonde urinaire ôtée après dix jours. L'examen histologique réalisé par deux centres différents d′anatomopathologie, conclut à la présence d′un kyste hydatique du sigmoïde dont la paroi est bordée par des membranes hydatiques sans signes de malignité (Figure 4). Avec un recul de 6 mois, le contrôle clinique ainsi que cystographique est normal.

## Discussion

Les fistules vésico-sigmoïdiennes (FVS) se définissent par l'existence d'un trajet anormal reliant le sigmoïde à la vessie. De point de vue anatomopathologique, on peut différencier les fistules entéro-vésicales sous péritonéales qui sont plus rares, post-traumatiques, iatrogènes ou secondaires à une pathologie tumorale pelvienne, des fistules entéro-vésicales transpéritonéales, fréquentes (60%) [1], habituellement secondaire à une sigmoïdite diverticulaire [2]. La formation de la fistule évolue classiquement par trois étapes successives: l’étape inflammatoire du sigmoïde suivie par la réaction péri sigmoïdienne avec formation d'adhérences inflammatoires serrées entéro-vésicales puis l'abcédassions se fistulisant dans la vessie. En raison de régime de pression élevé dans l′intestin, la fistule fonctionne le plus souvent de l′intestin vers la vessie sauf en cas d′obstacle sur le bas appareil urinaire [3]. Le tableau clinique est dominé au départ par les signes digestifs (troubles du transit, douleur hypogastrique..), associés parfois à des signes urinaires (60%) à type de brûlures mictionnelles, pollakiurie qui peuvent prendre le devant de la scène et faire penser à d'autres diagnostics, c’était le cas de notre observation. La phase de fistule constituée, peut se manifester par une pneumaturie et/ou fécalurie, qui sont pathognomoniques de fistules urodigestives mais ne sont présentes que dans 50% des cas [4].

Le diagnostic des FVS est posé habituellement par un faisceau d'arguments cliniques et paracliniques. Les examens radiologiques peuvent faire défaut, ainsi le lavement baryté n'objective la fistule vésico-sigmoïdienne que dans 25% des cas [5], Cet examen peut être complété par une colonoscopie qui pourrait être gênée par l'inflammation rendant toute biopsie aléatoire. Mais ces deux examens restent obligatoires dans le bilan d'une FVS pour écarter une malignité rectocolique sous-jacente [6]. L'urographie intraveineuse (UIV) a peu d'intérêt dans le diagnostic des FVS, mais garde son importance dans l’évaluation du haut appareil, toutefois elle peut montrer des signes indirects à type de niveaux hydroaériques dans la vessie, une encoche sur le bord supérieur de la vessie ou une fixité du dôme vésical. A noter que l'UIV est normale dans 80% des cas [7]. La cystoscopie garde une place importante dans le bilan des FVS, malgré qu'elle est sans particularité dans le quart des cas [8]; elle retrouve une zone inflammatoire polyploïde érythémateuse pseudotumorale autour de l'orifice fistuleux, comme c’était le cas chez notre patient sans mettre en évidence directement le trajet fistuleux. L'uroscanner est de grande valeur dans le diagnostic des FVS, il permet le diagnostic positif par l'opacification directe du trajet fistuleux et/ou la mise en évidence de bulles gazeuses dans la vessie, en plus il renseigne sur l’état du côlon et sur la présence ou non d'abcès intra abdominaux qui pourraient être ponctionnés par voie trans cutanée permettant ainsi une chirurgie à froid dans des meilleures conditions [9].

Une FVS constituée ne guérit pas ni spontanément ni avec un traitement médical, l'indication du traitement chirurgical est formelle, mais rarement en urgence. L'un des premiers traitements, préconisé en 1983 par Borrier et al. pour la FVS, consistait à effectuer une colostomie de décharge. Le traitement classique en trois temps (colostomie, colectomie, fermeture de la colostomie) a été popularisé dans les années 1930. Depuis les années 1950, plusieurs auteurs rapportent les excellents résultats obtenus en une seule intervention (colectomie, anastomose colo-colique immédiate sans colostomie de décharge) [10]. Puisque les FVS ne sont pas une urgence chirurgicale, le patient peut être opéré à froid. Le contrôle de l′état septique par les antibiotiques, la préparation colique, les progrès de la chirurgie et la modernisation de service de réanimation permettent de nos jours de traiter les fistules en un temps opératoire chez des patients sélectionnés.

Les FVS inflammatoires peuvent parfois être traitées par un traitement médical associant drainage vésical prolongé et antibiothérapie, essentiellement chez le sujet âgé en mauvais état général, et présentant une fistule entérovésicale bien tolérée. Le plus souvent, on procède à une colectomie segmentaire avec fermeture de la brèche vésicale, éventuellement interposition épiploique et drainage vésicale pendant 10 jours. Le rétablissement de la continuité sera réalisé en principe dans le même temps si les conditions locales (pas de collection abcédée péritonéale, préparation digestive) et générales (bon état général) le permettent. L′hydatidose intestinale n′a jamais été incriminée dans les fistules entéro-vésicales vue la localisation très rare de cette affection parasitaire au niveau de l′intestin qui semble passer le plus souvent inaperçue. Le traitement en un temps évitera non seulement le taux de mortalité et de morbidité liée à la fermeture secondaire de la colostomie, mais aussi permettra une meilleure qualité de vie au patient. [11].

## Conclusion

Les FVS secondaires à l'hydatidose sigmoïdienne sont exceptionnelles. Le tableau clinique reste dominé par la pneumaturie et/ou fécalurie, qui sont pathognomoniques. Le diagnostic est basé sur un spectre d'arguments clinique, paracliniques et essentiellement anatomopathologique. Le traitement est chirurgical après une bonne préparation générale et locale.
